# Influence of Provider and Leader Perspectives about Concurrent Tobacco-Use Care during Substance-Use Treatment on Their Tobacco Intervention Provision with Clients: A Mixed-Methods Study

**DOI:** 10.3390/ijerph20075260

**Published:** 2023-03-24

**Authors:** Maggie Britton, Isabel Martinez Leal, Midhat Z. Jafry, Tzuan A. Chen, Anastasia Rogova, Bryce Kyburz, Teresa Williams, Lorraine R. Reitzel

**Affiliations:** 1 Department of Health Disparities Research, The University of Texas at MD Anderson Cancer Center, 1400 Pressler Street, Unit 1440, Houston, TX 77030, USA; 2 Department of Psychological, Health & Learning Sciences, The University of Houston, 3657 Cullen Blvd Stephen Power Farish Hall, Houston, TX 77204, USA; 3 Health Research Institute, The University of Houston, 4849 Calhoun Rd., Houston, TX 77204, USA; 4 Integral Care, 1430 Collier Street, Austin, TX 78704, USA

**Keywords:** disadvantaged groups and tobacco-use disorder, substance-use disorders, concurrent treatment, provider perceptions, mixed methods

## Abstract

People with substance-use disorders have elevated rates of tobacco use compared with the general population, yet rarely receive tobacco-dependence treatment within substance-use treatment settings (SUTS). One barrier to delivering evidence-based interventions in SUTS is providers’ misconception that treating tobacco use and non-nicotine substance use concurrently jeopardizes clients’ substance-use recovery, although research indicates that it enhances support for recovery and relapse prevention. A total of 86 treatment providers employed in SUTS (i.e., 9 Federally Qualified Health Centers, 16 Local Mental Health Authorities (LMHAs), 6 substance-use treatment programs in LMHAs, and 55 stand-alone substance-use treatment centers) in Texas, USA, answered survey questions about their (1) thoughts about treating tobacco during substance-use treatment, and (2) delivery of the 5A’s tobacco-use intervention (Ask, Advise, Assess, Assist, Arrange). Twenty-six providers and leaders were interviewed about attitudes toward tobacco-free workplace policies and tobacco dependence and the relative importance of treating tobacco (vs. other substance-use disorders) at their center. Providers who did not believe tobacco use should be addressed as soon as clients begin treatment (i.e., endorsed responses of after 1 year, it depends on the client, or never) had lower odds of Asking clients about their tobacco use (OR = 0.195), Advising clients to quit smoking (OR = 0.176), and Assessing interest in quitting smoking (OR = 0.322). Qualitative results revealed barriers including beliefs that clients need to smoke to relieve the stress of substance-use recovery, are disinterested in quitting, fears that concurrent treatment would jeopardize substance use, and limited resources; additional training and education resources was the key facilitator theme. The results demonstrate a critical need to eliminate barriers to tobacco-treatment provision for clients in SUTS through education to correct misperceptions, specialized training to equip providers with knowledge and skills, and resources to build center capacity. Integrating evidence-based smoking interventions into routine care is key to support the recovery efforts of clients in SUTS.

## 1. Introduction

People with non-nicotine-substance-use disorders (e.g., alcohol-use disorder, opioid-use disorder) are a priority group for tobacco intervention [1,2,3,4]. Nationwide efforts in recent decades in the United States have successfully contributed to a reduced smoking prevalence of 12.5% among the general population [5]; however, people seeking treatment for non-nicotine substance use have not benefitted equally from these population-level efforts. In fact, rates of cigarette smoking among clients in alcohol and/or drug treatment remain unduly high, with many estimates ranging from 59% to 87%, and have been relatively stable over time, with little-to-no reductions realized [6,7,8,9,10]. As a result, people with non-nicotine-substance-use disorders suffer from elevated tobacco-related morbidity relative to the general population [11,12,13]. Given these steadfast disparities, there is both an opportunity and a need for targeted tobacco cessation efforts in substance-use treatment settings [14].

Historically, tobacco-cessation efforts have been limited in these settings, despite nationally issued clinical practice guidelines directing healthcare providers to address tobacco use among clients with non-nicotine-substance-use disorders (i.e., provide concurrent treatment) [15]. In Texas, 2016 data from mental-health and substance-use treatment centers reveal that only 48.9% and 64% (respectively) of centers were screening clients for tobacco use, and far fewer provided any treatment (47.4% offered counseling, 26.2% offered nicotine replacement therapy, and 20.3% offered non-nicotine tobacco-cessation medications) [16]. More recent data (2021) from a diverse range of Texas healthcare settings providing substance-use treatment suggest some potential improvement over recent years: 75.5% of centers mandated tobacco-use screening, 35.7% offered free nicotine replacement therapy, and 43.0% of providers reported helping clients who smoked cigarettes make a quit attempt through direct intervention [17]. Despite this promising upward trend in tobacco-cessation service provision, there remains much work to be carried out to reach clinical practice guideline standards.

Unfortunately, there is a longstanding tobacco-permissive culture within healthcare settings where substance-use treatment is provided. Pervasive beliefs among staff include that clients do not want to or cannot quit using tobacco [18], and that treating tobacco use will interfere with treatment for, or recovery from, other non-nicotine substances [18,19,20]. However, these beliefs are unfounded. Most clients in treatment for non-nicotine substance use want to quit using tobacco and have made multiple unsuccessful attempts to do so [10,20,21,22]. Furthermore, the treatment of tobacco use has demonstrated either a beneficial, or at worst, null, effect on other substance-use treatment and recovery [22,23,24,25,26]. Finally, clients support the provision of evidence-based tobacco-dependence treatment services in substance-use treatment settings; when these services are furnished, clients experience success in quitting tobacco [24].

Less than ideal beliefs have been identified as contributing to a reluctance to provide tobacco-dependence treatment in substance-use treatment settings [27]. However, only one study that we are aware of—published in 2007 with data collected between 2002–2004— empirically examines the association between providers’ attitudes and smoking-cessation treatment *availability*. Using surveys from 3786 staff (both providers and general staff) working within 348 diverse substance-use treatment settings (stand-alone substance-use treatment center, hospital or medical clinics, and government agencies such as Veteran’s Administration-affiliated clinics), this work found that support for integrating smoking cessation with non-nicotine-substance-use treatment was positively correlated with whether smoking cessation was offered at the center [28]. The present study builds on this prior work by statistically testing whether treatment providers’ attitudes toward concurrent smoking or tobacco cessation and non-nicotine-substance-use treatment are associated with their *provision* of an evidence-based, brief smoking-cessation intervention (i.e., behavior). Despite the paucity of extant work in this niche area (i.e., providers’ attitudes of concurrent treatment and their smoking-cessation intervention behavior), this work is theoretically supported by a large amount of literature (i.e., hundreds of publications), stemming from The Theory of Planned Behavior (TPB) [29]. The TPB posits that attitudes toward behaviors are strong predictors of actual behavior and has been applied broadly across healthcare services work [30]. The present study uses the 5A’s (Ask about tobacco use, Assess their desire to quit smoking, Advise them to quit smoking, Assist those who have a desire to quit to access treatment resources, Arrange a follow-up session to check in on their progress) as an assessment of provider behavior [15,31]. This brief intervention (i.e., the 5A’s) is recommended by the *Treating Tobacco Use and Dependence: Clinical Practice Guideline* as an evidence-based practice for smoking cessation [15]. Furthermore, the present study uses a mixed-methods design, bringing in qualitative data to elucidate on provider perspectives about treating tobacco use and better explain the quantitative findings.

## 2. Materials and Methods

### 2.1. Study Design

**Figure 1:**
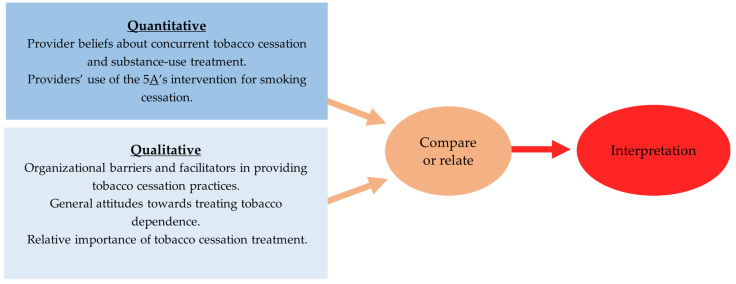
Convergent Mixed-Methods Design.

A convergent mixed-methods design was adopted for this study, which focused on how providers’ beliefs regarding concurrent tobacco- and substance-use disorders impact the delivery of tobacco interventions to clients (see Figure 1). Quantitative data assessed the impact of providers’ beliefs about concurrent smoking cessation and non-nicotine-substance-use disorder treatment on the delivery of the 5A’s; qualitative data provided insight into the contextual factors and processes that constrain or enable providers in the provision of tobacco interventions more broadly. The results from the two datasets were merged through comparing and synthesizing the quantitative results on outcomes and qualitative findings on context and processes [32]. Quantitative and qualitative data were collected and analyzed independently by respective analysts and merged during the final interpretation. The purpose of the mixed-methods design within this study was for *complementarity*—in which qualitative findings are used to elaborate upon quantitative results to yield a more complete understanding [33].

### 2.2. Procedures and Participants

From April to December of 2021, survey and interview data were collected as part of a statewide needs assessment of Texas healthcare centers providing non-nicotine-substance-use care—including Federally Qualified Health Centers (FQHCs), Local Mental Health Authorities (LMHAs), dedicated substance-use treatment programs within LMHAs, and stand-alone substance-use treatment centers—to discern organizational practices and provide perceptions of their clients’ tobacco use.

The research team, with the help of community liaisons, solicited contact information for healthcare centers and pursued recruitment through either email or postal mail. Moreover, recruitment was facilitated by team members attending professional organization meetings/conferences as a vendor where they promoted the needs assessment. The team additionally received recruitment support from the Texas Department of State Health Services, the contractor of the needs assessment, through presentations in meetings of the Tobacco Prevention and Control Coalitions, regional coordinator meetings, and Community Resource Coordination Groups in Texas. Lastly, recruitment was aided by professional organization leaderships who shared member lists or promoted the needs assessment through their Listservs.

A cover letter explaining the aim of the study with critical elements of informed consent preceded an electronic Qualtrics survey. Moreover, the cover letter also described that the respondents would be compensated with a USD 20 Amazon gift card upon completion of at least 75% of the survey. Targeted respondents included representatives from identified Texas FQHCs (n = 57), all global LMHAs (n = 39), identified substance-use treatment programs in LMHAs (n = 89), and identified stand-alone substance-use treatment centers (n = 458) across the state who knew about the organization’s tobacco-use policies and practices. The aim was to receive 1 completed or near-completed survey per healthcare center, whether by a direct treatment provider or other staff members who did not provide direct client treatment/care. After the removal of duplicate surveys (n = 10, representing 5 centers), response rates were as follows: 43.9% of FQHCs (n = 25/57), 76.9% of global LMHAs (n = 30/39), 15.7% of substance-use treatment programs within LMHAs (n = 14/89); and 14.4% of stand-alone substance-use treatment centers (n = 66/458). Due to the specific focus of this study, we were interested only in respondents who indicated that they provided substance-use treatment to adults at their organization (N = 86), which included 9 FQHCs, 16 global LMHAs, 6 substance-use treatment programs in LMHAs, and 55 stand-alone substance-use treatment centers.

Upon survey completion, respondents could indicate their interest in further study participation via completing a virtual interview. Forty-eight respondents indicated interest in, and were solicited for, participation in an interview. For the qualitative sample, purposeful sampling was used to select interview participants from among all survey respondents (N = 125)—either those providing direct services to clients, or leaders and managers—who were knowledgeable regarding the current tobacco-use policies and procedures of their organizations. Criteria for selection included interviewing at least 1 participant in each of the 11 Public Health Regions in Texas [34]. A total of 26 participants were interviewed, representing 10 Public Health Regions. Participants were compensated with an additional USD 25 electronic gift card for interview participation. Of the 26 participants, 10 were counselors, and 8 were organizational leaders or departmental managers who also provided direct services to clients, and 8 were managers who were not involved in client direct service provision. As such, the qualitative sample represents the views of both direct service providers as well as organizational leaders and departmental managers. Because the present project did not meet the definition of human subject research per the University of Houston’s Institutional Review Board (IRB), IRB approval was not needed.

### 2.3. Quantitative Measures

#### 2.3.1. Healthcare Center Characteristics

Descriptive characteristics of healthcare centers included: (1) the type of healthcare center; (2) the number of unique clients seen annually (later categorized based on sample distribution as 50–200, 201–1000, and >1000; (3) the number of full-time employees (later dichotomized based on sample distribution as 1–50 vs. >50); and (4) whether the center had a comprehensive tobacco-free workplace policy, defined as prohibiting tobacco use both indoors and in all areas of the center’s property (yes vs. no).

#### 2.3.2. Providers’ Beliefs about Concurrent Tobacco Cessation and Substance-Use Treatment

Providers of substance-use treatment were queried with two items for their beliefs on concurrent tobacco-cessation and non-nicotine-substance-use treatment. Beliefs about smoking cessation were queried more directly because cigarettes are still the most used tobacco product by adults in the United States [35]. First, respondents answered the following item: “In your opinion, what is the best point to encourage clients to stop smoking or using other tobacco products?” Options included: (1) “as soon as they begin treatment”; (2) “after 1 year of treatment at your organization”; (3) “it depends on the client”; and (4) “never”. For analytic purposes, responses of “as soon as they begin treatment” were compared with all other responses collectively, given that cessation as soon as treatment begins is the best clinical approach [23,36]. Second, given that alcohol-use disorder is the most prevalent non-nicotine-substance-use disorder in the United States [37], providers were asked to rate their level of agreement with the following statement: “If a client has been in recovery from alcoholism for <6 months, quitting smoking would jeopardize their recovery”. Each item was rated using a 5-point Likert scale ranging from strongly disagree to strongly agree; endorsements of strongly disagree or disagree were compared with neither agree nor disagree, agree, or strongly agree to make practical comparisons between providers who disagreed with the statement (i.e., indicating more favorable views toward concurrent treatment) and those who were ambivalent toward or contested the statement (i.e., indicating less favorable views toward concurrent treatment).

#### 2.3.3. Providers’ Use of the 5A’s Intervention for Smoking Cessation

Providers were asked to report on their use of the 5A’s intervention for smoking cessation within the last month. Questions queried included how frequently the provider: (1) Asked clients about their current use of cigarettes; (2) Advised cigarette users to quit; (3) Assessed cigarette users’ interest in quitting; (4) Assisted cigarette users through on- or off-site referrals (including to the Texas Tobacco Quitline) and/or through direct intervention; and (5) Arranged a follow-up appointment to assess progress with the quit attempt. Each item was rated using a 5-point Likert scale ranging from never to always. Similar to previous research and in order to facilitate comparison, endorsements were grouped as follows: never/sometimes/about half the time vs. most of the time/always [38,39,40,41,42,43,44,45].

### 2.4. Qualitative Procedures

Research aims guided the development of a semi-structured interview guide that was tested and adapted according to participants’ responses in the field. Interviews took place virtually from April–November 2021, were audio and video recorded following provider’s consent, and lasted 35–60 min. Interviews were conducted by an anthropologist and public health researcher (I.M.L.) and a cultural anthropologist (A.R.) both trained in qualitative research. Interview questions focused on the main barriers and facilitators organizations had faced in providing tobacco-cessation practices, general attitudes towards treating tobacco dependence within their organization—among providers and organizational leaders and the relative importance of tobacco-cessation treatment to participants as substance-use providers.

### 2.5. Data Analysis

Data were reported with descriptive statistics. Logistic regression analyses, controlling for healthcare center type (given differences in tobacco screening/treatment rates), and comprehensive tobacco-free workplace policy (given associations with screening/treatment rates) [16,46], were conducted to assess the associations between the dependent variables (respondent’s smoking-cessation intervention practices with clients; i.e., 5A’s) and the independent variables (beliefs about concurrent smoking/tobacco cessation and treatment of non-nicotine substances). The analyzable sample was 83 providers due to missing data on one or more of the variables of interest for 3 respondents. The significance level was designated at *p* < 0.05. All analyses were conducted using SAS version 9.4.

Interviews were transcribed verbatim by a professional transcription service and uploaded onto Atlas.ti9 (Atlas.ti, version 9.1.7, Berlin, Germany, 2020) to organize data analysis. Coding proceeded iteratively, using thematic analysis and constant comparison to continually compare emerging and new data to previously coded transcripts in order to systematically identify, code, and categorize the key patterns or themes in the data. Two analysts, (I.M.L.) and (A.R.), independently coded preliminary transcripts and met regularly to reconcile any coding discrepancies to develop an initial coding frame. The coding frame remained flexible to refinement so that new codes could be added with the analysis of successive interviews, until no new codes emerged [47]. The final coding frame was reapplied to all the data. Through the process of constant comparison of the data, codes were combined and synthesized into themes that were drawn directly from the dataset and refined through analyst triangulation to ensure congruency and credibility of findings. Pseudonyms were used throughout this article to respect participant privacy and confidentiality [48].

## 3. Results

### 3.1. Quantitative Results

#### 3.1.1. Healthcare Center Characteristics and Provider Information

Table 1 provides a summary of responding providers’ healthcare center characteristics, providers’ perceptions on concurrent smoking/tobacco cessation and non-nicotine-substance-use treatment, and providers’ smoking-cessation intervention practices (i.e., provision of the 5A’s) with clients. Chi-square/Fisher’s exact tests between healthcare center characteristics and provider information are presented in the Supplemental Files, Tables S2–S5.

#### 3.1.2. Logistic Regression Analyses

Logistic regression analyses (presented in Table 2), adjusted for healthcare center type and comprehensive tobacco-free workplace policy, revealed that providers who did not believe tobacco use should be addressed as soon as clients begin treatment (i.e., endorsed responses of after 1 year, it depends on the client, or never) had lower odds of Asking clients about their tobacco use, Advising their clients to quit smoking, and Assessing their interest in quitting smoking. Providers’ perceptions that quitting smoking jeopardizes recovery from alcoholism was not significantly related to their intervention practices. Unadjusted models are additionally presented in the Supplemental Files (Table S6).

### 3.2. Qualitative Findings

Analysis of interview transcripts yielded four main themes influencing the provision of tobacco-dependence interventions, including the 5As, among clients (Table 3): (1) Misconceptions about the concurrent treatment of tobacco and substance use: smoking reduces stress and is a valuable coping mechanism; (2) “Blame the victim” mentality: clients are not interested in quitting smoking; (3) attitudes towards smoking cessation: (a) devaluing of tobacco dependence as a serious addiction, (b) clients were overwhelmed by multiple social inequities and did not have the wherewithal to quit smoking; and (4) limited resources for addressing tobacco dependence. Each theme is described below and illustrated using participants’ direct quotes. While interview questions asked about tobacco use, in the quotes below, providers and leaders mostly refer to smoking, given its prevalence among their clients and the fact that smoking constitutes tobacco use and is the most common form of tobacco use and tobacco dependence [35]. As such, participants often used the term interchangeably below.

#### 3.2.1. Misconceptions about the Concurrent Treatment of Smoking/Tobacco and Substance Use: Smoking Reduces Stress and Is a Valuable Coping Mechanism

Many providers and leaders reported misconceptions regarding the concurrent treatment of smoking/tobacco and substance use that unfortunately remain prevalent within substance-use treatment settings. The idea that smoking tobacco reduces stress and serves as a valuable coping mechanism for substance-use disorder clients is a widespread myth within these treatment centers. Participants reported support within their center for the idea that clients need to smoke to offset the increased pressure and anxiety created by their recovery efforts from other substance dependency:

*I think a lot of our staff though, especially the ones who are tobacco users, they tend to approach tobacco use in conjunction with substance use as an issue of harm reduction. So, if somebody is trying to quit using one substance, they try to make the argument that you shouldn’t be trying to quit using tobacco at the same time because it’s stressful. We’ve tried to train our staff to understand that’s a misconception and the research does not back that up.*

(Lisa, direct service provider, Director of Operations)

*The clients, they’re dealing with enough as it is. They’re dealing with a lot as far as addiction. You know, they’re trying to get off of drugs and alcohol. That’s hard enough as it is, let alone trying to quit smoking. That’s their vice. I mean, I understand that. If we had a smoke-free environment, I probably wouldn’t have a job. [Laughter] You know, I teach the smoking cessation programs.*

(Martha, direct service provider, Quality Mental Health Professional, Tobacco Treatment Specialist)

Some providers and leaders acknowledged that these misconceptions were part of the culture of substance-use treatment, but felt that they could be addressed with adequate training:

*I think more training is needed. I also think there’s a stigma there where mental health clients, they’re smoking and it calms them down and it’s just happened a lot, especially with the higher levels of care where our staff—I’ve heard some staff say, “They need to smoke. They need to smoke because if not, they get anxious. Keep on giving them cigarettes.”… There’s that connection with addiction and also just giving in to that behavior and not working with the client. I think that’s something that’s a part of our culture—at least in the area that could change, not really just being okay with it—I think it’s something that we can improve on. I’m talking about the community in general.*

(Robert, direct service provider, Licensed Professional Counselor/Chemical Dependency Counselor)

#### 3.2.2. “Blame the Victim” Mentality: Clients Are Not Interested in Quitting Smoking Tobacco

“Blame the victim” is an attitude that has been learned by health care providers in working with various health conditions, commonly associated with stigma, e.g., smoking tobacco, substance use, and obesity, in which rather than contextualizing the development of a health condition within the larger socioeconomic context, the individual is held responsible for their condition [49]. While a few providers and leaders reported that their clients had a genuine desire to quit smoking tobacco, most stated that clients were not interested in quitting, and therefore, providers did not pursue addressing their tobacco use with them:

*They [clients] really don’t want to quit, and there’s really nothing to tie the action to in terms of a reward for a lot of them. So, they don’t see the benefits, or they don’t care about improving their health. That’s not what they’re focused on. So, if there’s no reward like in terms of an incentive or a financial reward, they don’t follow through.*

(Edith, Director of Behavioral Health Care Services)

However, while citing a lack of client interest in quitting smoking tobacco as a primary reason for not offering tobacco-cessation interventions, some providers and leaders did acknowledge that their actions were complicit in this reluctance to treat tobacco dependence:

*The barrier [to providing tobacco cessation] would be the clients not really being interested in stopping the use of tobacco, being more focused on their other substances or the clinicians and the case managers and peer coaches making sure that they find time to include that as part of somebody’s recovery plan or treatment plan.*

(Lisa, direct service provider, Director of Operations)

Additionally, providers and leaders shared that it was up to the client to initiate the receipt of tobacco-dependence treatment, rather than providers offering these services to clients. Some providers and leaders recognized that adopting a client-led process for receiving tobacco-cessation services essentially served as a hindrance to the receipt of such care:

[Tobacco cessation] *is client-directed, client-driven, client-volunteered…The counselors, the education process, the group processing, we talk about it, but we leave it totally up to the client to ask direction as to whether or not they want to try that…. Probably effort on my part and making it less of a client-driven opportunity; more of a staff-driven opportunity [would facilitate tobacco cessation treatment provision]. We can do better in terms of more than just “Here it is if you want it.” It’s more of a “Here’s an opportunity. Let’s show you how this would work.”*

(Frank, direct service provider, Executive Director)

#### 3.2.3. Attitudes towards Smoking/Tobacco Cessation

Providers’ and leaders’ attitudes towards smoking/tobacco cessation were a crucial factor influencing whether or not they would offer these services to their clients. There were two sub-themes that emerged within our analysis of this theme: (i) devaluing smoking tobacco as a serious addiction; and (ii) clients were overwhelmed by multiple social inequities and did not have the capacity to quit smoking tobacco.

i. Devaluing smoking tobacco as a serious addiction

The most prevalent attitude expressed by providers and leaders was that treating smoking/tobacco addiction was considered secondary to drug and/or alcohol addiction; as such, it was either not addressed or was ostensibly deferred to a later time (that generally never materialized):

*There is sort of this mentality here, “Let’s take care of the most serious addiction first and let’s talk about the other pieces of your addiction as you go along.” We try to deal with one addiction at a time.*

(Frank, direct service provider, Executive Director)

Additionally, while all healthcare centers that provide substance-use treatment in Texas are mandated by the state to conduct smoking/tobacco screenings of clients, some providers and leaders expressed the view that these screening procedures were less than adequate; clients were asked if they used tobacco but not if they wanted to quit smoking or using other tobacco products. These participants were aware that the tobacco screening procedures that were being used at their center represented the bare minimum and needed to be improved.

*It’s* [tobacco screening] *just included in the substance use history. It’s really just asking “What type of tobacco product or nicotine product do you use, what was your age of first use, how much you currently use and do you have any abstinence history?” That’s about it… I certainly have already thought about talking to our vice president, our clinical person about adding a piece there. Now, it would have to be very, very brief but at least we could just ask if they are interested in stopping* [tobacco use].

(Jane, direct service provider, Director of Step One Services)

ii. Clients were overwhelmed by multiple social disadvanatges and did not have the capacity to quit smoking tobacco

Providers and leaders noted that the clients they served were struggling with multiple intersecting social disadvantages—substance use, mental illness, unemployment, lack of medical insurance, insecure housing, and economic hardship. The providers’ and leaders’ perspective was that they should assist clients in addressing these significant issues first, as they were more acute or urgent, and then clients would be approachable and capable of tackling smoking/ tobacco cessation:

*I think that the biggest barrier is that folks that we serve have so many other major issues. When you don’t have a job or you don’t have appropriate housing or you don’t have food to feed your family or any of those things….Yes, you can argue, “Well, if you quit smoking, that would be money that you could use for food,” but when there’s so many overwhelming barriers in one individual’s life, that’s the last of the things that they’re going to focus on. So that, I think, is a huge barrier. We’re serving the indigent, low-income, uninsured population with lots of big issues, and so if you can …. help them resolve some of those other issues, what my staff tell me, then you can approach them about stopping smoking.*

(Edith, Director of Behavioral Health Care Services)

#### 3.2.4. Limited Resources for Addressing Tobacco Dependence

Most providers and leaders noted that underlying center limitations or barriers to addressing tobacco dependence among their clients was a lack of resources in various areas, including targeted training, funding, time, and staff. The receipt of no or limited specialized training to treat tobacco dependence was a key theme noted by many participants; one felt to be crucial in improving organizational capacity to address client tobacco use, especially within the context of treating tobacco and substance use simultaneously:

*Well, first of all, I think there has to be a lot of education on the link between continuing smoking and risk of relapse. There would have to be that buy-in for that piece of it because if there’s not—and again, it’s very old school and I know it’s antiquated but I think it’s very—I don’t think I’ll employ the only person in the treatment realm or in recovery realm that feels like you shouldn’t quit all at once. That’s kind of has always been the thought process. “Okay. Well, you’re quitting. Your three or four drugs of choice were—or we definitely don’t want you to quit smoking.” I mean that’s definitely been an old-school mentality. I think that education would be a key.*

(Jane, direct service provider, Director of Step One Services)

Consequently, providers and leaders expressed an interest in receiving additional training, and noted the value of training in increasing provider buy-in on the necessity to provide clients with smoking-/tobacco-cessation interventions:

*[…] that [tobacco education training] would probably be an area of improvement for us to provide better clinical training for tobacco use and cessation for our clinical staff. That might help bring them onboard more actively to encourage people to try to quit using tobacco if they had a better understanding of that […] I think that the clinicians probably need more training and to create better buy-in on the importance of not using tobacco products. Also encouraging their clients and participants to pursue tobacco cessation as well as recovery from other substances.*

(Lisa, direct service provider, Director of Operations)

Understandably, limited financial resources played a major role in constraining providers’ and leaders’ ability to provide tobacco-cessation services, being the foundation upon which additional staff, and the related factor of time, depended:

*I think it all comes down, I guess, in the bottom line, it all comes down to funding. Like I said, funding in the form of being able to dedicate specific staff to just this issue, to run smoking cessation groups, to do the trainings. I think what we’re doing is pretty much—well, maybe it’s not the bare minimum […] but there’s so much potential for this to grow but it all comes down to lack of staff, which comes down to lack of resources to be able to hire and train staff and have them specifically devoted to that.*

(Susan, Director of Special Programs)

This participant went on to note the harmful impact of this lack of resources on providing clients with needed care and services:

[Providers’] *case load is high and they have limited time with people and that not an environment that is conducive to prioritizing smoking cessation, let me just put it like that. It’s like, I’m supposed to see now my time cut down. Now my time is cut down by this many minutes to see this many clients, and now we have two vacant positions and so it’s just nonstop, nonstop, nonstop. My groups are growing bigger. The timeframe that I have to meet with people is growing smaller and it’s just—you tend to get on that slimmed down version of service provision.*

(Susan, Director of Special Programs)

## 4. Discussion

This work leveraged a mixed-methods approach to assess how substance-use treatment providers’ perceptions of concurrently treating tobacco use and non-nicotine substance use impacted tobacco-use intervention in practice. Specifically, the association between provider attitudes toward concurrent treatment and the provision of the 5A’s for smoking cessation was statistically tested, and findings revealed that providers who endorsed that smoking/tobacco cessation should be encouraged after 1 year of treatment at the organization, never, or depending on the client, were less likely to Ask, Advise, or Assess clients’ interest in quitting smoking (compared with those who said as soon as treatment begins). The qualitative component investigated providers’ and organizational leaders’ beliefs and attitudes towards concurrent treatment, yielding a deeper understanding of the larger context and issues that impact providers’ provision of tobacco-cessation interventions. These findings reveal that provider attitudes can have direct and negative implications for client care. Research supports that smoking/tobacco cessation should be addressed as soon as treatment begins (a fact that only 39.76% of the providers endorsed) to best support clients’ positive treatment outcomes and recovery [23]. Most clients who smoke cigarettes and are receiving care in substance-use treatment settings want to quit [21], and people who smoke cigarettes make, on average, 30 quit attempts before finding success, indicating that there is an opportunity and need to intervene both early and often [50]. Therefore, it is critical to work toward shifting provider and organizational leader perceptions through training and resource provision to support tobacco intervention urgency.

Unfortunately, most providers (57.83%) felt that the point during treatment at which to address a client’s smoking/tobacco cessation should depend on the client. In line with this, the qualitative data revealed that providers and organizational leaders held misperceptions that clients did not want to quit smoking (implying that unless a client wanted to quit, they should not intervene). Qualitative findings on provider and leader misconceptions about the concurrent treatment of tobacco and substance use included the belief that smoking reduces stress, and therefore, serves clients as a valuable coping mechanism. Participants reported that not addressing tobacco and substance use concurrently was seen by many providers as an effective means to reduce client harm, meaning they felt that asking clients to quit tobacco use simultaneously with substance use would cause more harm than good. These beliefs effectively serve to limit providers’ delivery of tobacco-control interventions, which are supported by quantitative results on lower rates of Asking, Advising, and Assessing clients’ interest in quitting smoking. However, as Prochaska (2010) noted, adopting the position that not treating tobacco dependence within mental-health and substance-use treatment settings constitutes harm reduction is misguided [51], and on the contrary, can contribute to client harm because tobacco use: (1) is the leading cause of death in clients with addictive and psychiatric disorders [52]; (2) is linked to worse substance-use treatment outcomes, whereas the treatment of tobacco dependence is associated with long-term abstinence from alcohol and other substances [25]; (3) is linked to increased depression, anxiety, and stress [53]; (4) is not an effective long-term coping strategy to manage stress; (5) treating tobacco dependence does not adversely impact mental health or substance-use recovery [51]; and (6) harms others through second-hand smoke.

Qualitative analysis also identified that providers’ and leaders’ attitudes indicated a devaluing of tobacco dependence as a serious addiction requiring intervention and that clients had more serious and acute issues—i.e., substance use and other pressing issues related to social disadvantage that required immediate attention. Clients’ other “major issues” needed to be addressed first (vs. tobacco/smoking, which was considered a secondary or more minor concern)—even though the high cost of cigarettes were an additional burden exacerbating clients’ challenges. The implication was that once these more pressing, acute issues had been addressed, then providers could address tobacco dependence with clients. However, it stands to reason that this deferment of tobacco-dependence treatment within these settings results in clients never receiving assistance with quitting tobacco use, as most residential programs are 90 days in duration. Research comparing studies that used a concurrent vs. delayed or waitlist treatment design within substance-use treatment settings has shown that between 23 and 100% of the deferred group never did receive a tobacco-cessation intervention [23]. Quantitative results also support this finding, as the majority (60.24%) endorsed that the best point to encourage clients on smoking/tobacco cessation was “after 1 year/it depends on the client/never”, suggesting the likelihood that deferring treatment to a future time equated to a failure to deliver a tobacco intervention. Coupled with the attitude that treating tobacco is devalued as secondary to the treatment of other non-nicotine substance uses, the prospect of providing clients with tobacco-cessation interventions, concurrently or otherwise, is unlikely. Recent research on addressing smoking cessation within these settings in Texas supports this finding, where 20.3% offered non-nicotine tobacco-cessation medication, and 43% directly intervened with clients to quit smoking [17]. The notion that dependence on tobacco is a “less serious addiction” than substance-use dependence is not supported by evidence. On the contrary, research shows that in 2019 in the US, tobacco-related deaths accounted for 549,585 deaths compared to 170,453 deaths related to illicit and licit drug use [54]. According to the Centers for Disease Control and Prevention, the mortality rates for people who smoke in the US are roughly 3X higher than among similar individuals who never smoked, and life expectancy is lowered by at least 10 years [55].

There is no doubt that clients who are experiencing the deprivation of homelessness [56] or living on very low incomes in the United States require additional and sustained assistance on multiple levels to support them in staying abstinent from all substance use, including tobacco, given the additional social and health inequities borne by these groups such as social stigma, lack of health insurance, and access to regular medical care and cessation therapies. The higher smoking rates among those experiencing disadvantage has long been recognized, leading to the acknowledgement of smoking as a social justice issue [57,58,59,60]. Addressing the multiple and complex needs of these subgroups living with disadvantage requires comprehensive nicotine and non-nicotine-substance-use treatment interventions that target the multi-level factors contributing to substance use among these groups, including population-level interventions [56,61,62,63]. Reviews of tobacco-control interventions indicate that systemic changes are needed to effectively address smoking cessation among disadvantaged communities, and that multi-faceted approaches as well as tailored interventions are more successful [45,64,65,66].

In interviews, providers and organizational leaders also reported two related client factors which they cited as contributing to their limited provision of smoking-/tobacco-cessation interventions: a lack of client interest in quitting smoking/tobacco use and relying on clients to initiate any tobacco-related interventions. While a few participants relayed that their clients were interested in quitting, most reported that clients had no desire to quit smoking/tobacco use either because they were more focused on substance-use recovery or did not see any benefits in cessation. In this study, this perspective was characterized as “blame the victim”, an attitude that has unfortunately been learned by many health care providers, particularly in regard to addressing stigmatized conditions such as tobacco and substance use and obesity, in which clients are often viewed as being responsible for having caused their condition, rather than contextualizing health behaviors [49]. However, a few did acknowledge that as substance-use providers and organizational leaders, they played a role in failing to deliver tobacco interventions to clients due to not making time to incorporate these services into recovery and treatment plans. Additionally, in reporting that the initiation of smoking-/tobacco-cessation treatment was dependent upon clients, some providers and leaders recognized that they needed to instigate and facilitate the provision of these services to clients. These instances of providers and leaders noting and reflecting upon missed opportunities to assist their clients with smoking/tobacco cessation is a promising finding, indicating an interest in changing their practice. However, there are also brief tobacco-use interventions that providers can deliver to motivate clients to consider a quit attempt, such as the 5R’s (Relevance, Risks, Rewards, Roadblocks, Repetition) intervention [67]. Tobacco-use disorder is recognized as the most common substance-use disorder in the United States [68]. As substance-use treatment professionals, allowing clients to initiate discussions about smoking/tobacco cessation is to passively hinder tobacco-dependence treatment. This finding also reinforces quantitative results indicating that most providers endorsed “it depends on the client” as the best time to encourage clients to quit smoking, thus impeding the delivery of life-saving tobacco-cessation interventions.

Providers and leaders also reported that a major barrier to addressing client tobacco dependence was a lack of resources across different areas, including targeted training, funding, time, and staff. Most providers and leaders reported that they had either not received any or limited specialized training to treat tobacco dependence, which was frequently cited as a key reason underlying lack of provision of cessation services to clients. Many providers and leaders cited the importance of receiving specialized provider training to building organizational capacity to treat tobacco dependence, particularly given the benefits of treating tobacco and other substance use concurrently, signaling that some providers were aware of the benefits of concurrent treatment. A lack of specialized training has been widely recognized as a primary barrier to the provision of tobacco-cessation treatment [69,70]. Limited financial resources to fund the hiring of additional staff to allow providers additional time to address tobacco cessation with clients is also a commonly cited barrier, as is high staff turnover [71,72]. Together, the lack of resources in knowledge, staff, and time support can also help explain the quantitative results on why providers engaged more in Asking, Advising, and Assessing, rather than Assisting and Arranging for smoking cessation. Therefore, it is important to educate providers and organizational leaders that tobacco use is a serious and deadly addiction that their clients are more likely to die from (vs. their non-nicotine substance use) and that it should be *addressed* as soon as treatment begins [54]. Meanwhile, it can be stressed that this does not equate to forcing clients to quit tobacco if they do not want to. Offering clients tobacco-cessation services is not coercion, research indicates that simply discussing cessation options with clients increased the likelihood that they will make a quit attempt [73,74]. Instead, it means having the infrastructure and capacity in place to identify every tobacco-using client and provide appropriate support and treatment to those who want to quit. Additionally, educating providers and organizational leaders through specialized trainings on treating tobacco use among clients with substance-use disorders would help correct the many misconceptions that exist within these treatment settings regarding clients’ ability and desire to quit smoking and concurrent treatment, as well as unlearning inherited passive attitudes towards treating clients’ tobacco dependence that puts the onus on clients rather than providers to lead these efforts. Significant research indicates that quitting smoking concurrently with other substance use is associated with 25% increased abstinence from alcohol and other substance use [23], while a more a recent study showed decreased consumption, decreased relapse, and increased past-year abstinence for alcohol and other substances [75]. As such, given the benefits of treating tobacco and other substance use concurrently, providers are not only missing an opportunity to assist their clients with quitting smoking, but hindering their substance-use recovery.

One possible explanation for null findings for Assist and Arrange is that providers are not equipped with the skillset to Assist in smoking cessation or are aware of the importance of Arranging follow-up care (given the many attempts it takes to successfully quit, and high relapse rates); in this case, even if providers feel that encouraging smoking/tobacco cessation as soon as treatment begins is important, there may be no association between this attitude and provision of care because they do not have the capacity to perform these steps, given their lack of specialized training. Furthermore, providers’ perceptions on the role of smoking cessation on alcohol-use recovery was not significantly associated with the provision of the 5A’s intervention. However, it is important to recognize that nearly half (45.78%) of providers agreed or strongly agreed that quitting tobacco *does* jeopardize alcohol-use recovery, despite robust scientific evidence to the contrary [23]. Provider education should be delivered in substance-use treatment settings to correct some of these concerning and highly endorsed misperceptions.

Despite the aforementioned strengths and novelty of this work (e.g., testing of a specific provider intervention, mixed-methods design), there are notable limitations as well. First, the data were pulled from a cross-sectional survey; therefore, it is impossible to state with certainty that provider attitudes caused the provision of the 5A’s. It is likewise possible that the providers’ provision of the 5A’s was responsible for or reinforced attitudes. Second, despite that the assessment of providers’ provision of an evidence-based brief intervention for smoking cessation is a notable strength of this work, the assessment of only one (i.e., the 5A’s) intervention is a limitation as it may not be representative of other interventions taking place within these centers. As such, future work should address additional interventions, such as the provision of pharmacotherapy, especially considering consistent null findings for Assist and Arrange. However, it is also worth noting that qualitative data suggest that little-to-no intervention is provided (aside from Ask and external referrals. Referrals can be powerful resources for providers who are short on time; however, they are likely to be ineffective without directly “connecting” clients to the referral site/source) [76,77], and therefore, such work may not reveal novel insights. Third, the provision of the 5A’s intervention was self-reported and subject to bias, including recall and social desirability bias. For a precise accounting of the delivery of these interventions, research could consider pulling from client records (e.g., electronic health record, paper chart); however, centers treating individuals with substance-use disorders may have little capacity (i.e., time) to do so. Fourth, and finally, the questions pulled from the quantitative needs-assessment survey were not designed to test the association in question, leaving significant room for methodological improvement. An example of this is that this work queried provider attitudes on the interference of smoking cessation with recovery from alcoholism, despite that it is unknown whether responding providers treated individuals with alcohol-use disorders (e.g., providers at opioid treatment centers). However, it is notable that alcohol-use disorder is the most prevalent non-nicotine-substance-use disorder, it co-occurs with other non-nicotine-substance-use disorders (i.e., 16% of individuals with an illicit substance-use disorder also have an alcohol-use disorder), and therefore, substance-use treatment providers’ (e.g., licensed chemical dependency counselors) attitudes toward concurrent smoking cessation and alcohol-use disorder may serve as a proxy for their general attitudes toward non-nicotine-substance-use treatment. It is likewise possible that this limitation to methodology explains the non-significant findings for this question. A second example of an area for methodological improvement includes that the survey questions generally asked about smoking cessation (or, smoking/tobacco cessation) and the qualitative interviews asked about tobacco cessation more broadly. A third area for methodological improvement includes that because the focus of the needs assessment was primarily on organizational practices, policies, etc., there was very little individual-level data collected on the providers (e.g., length of employment, race/ethnicity, age, gender, etc.) that could be helpful to contextualize findings. However, providers and leaders mostly refer to smoking in the interviews given its prevalence among their clients. Regardless, the fact that we cannot precisely tease apart attitudes toward smoking cessation vs. tobacco cessation is a limitation to the work. Future studies could strengthen this work by expanding to additional tobacco-use interventions (as mentioned above) but also to address provider attitudes about the potential for quitting tobacco to interfere with other substance-use (i.e., in additional to alcohol) treatment as well. Alcohol use, like tobacco use, is a legal drug, and providers may have varied perceptions on the possible interference for non-legal drug use. Additionally, future studies should leverage more constructs in the TPB to better understand factors that are driving providers’ inaction on tobacco-use treatment in these settings [29]. For instance, assessing providers’ *strength of behavioral beliefs* related to the concurrent treatment of tobacco use and non-nicotine substance use could provide nuanced insight into predicting provider treatment behaviors. Items that could assess this might include: “If I treat tobacco use, I will prevent future problems for my patients” or “If I treat tobacco use, I will feel that I am doing something positive for the patient”. Additionally, norms are powerful drivers of behavior. Therefore, assessing providers’ *subjective norms* related to concurrent treatment might help elucidate on normative pressures influencing behavior. Items that could assess this might include determining providers’ agreement with the statements: “Patients with substance use disorders expect providers’ to treat their tobacco use” or “Other providers treat tobacco use among patients with substance use disorders” [30].

## 5. Conclusions

Each of the main themes of the qualitative analysis is congruent with and supports the quantitative results. Together, these findings reveal key aspects of providers’ and organizational leaders’ attitudes towards concurrent treatment that impact their provision of tobacco intervention practices and are aligned with previous studies on the beliefs and perspectives of providers towards tobacco cessation [20,78,79,80]. In using a mixed-methods approach, the study findings extend this prior research by combining qualitative findings on the beliefs, attitudes, and contextual factors that can facilitate or constrain providers and organizational leaders in concurrently treating tobacco- and substance-use dependence, and quantitative results that assessed the impact of these beliefs on their delivery of the 5A’s to clients. Combining the analyses from both components yielded a more comprehensive understanding than either method could singularly [81] of the challenges facing substance-use providers and organizational leaders in addressing tobacco dependence among their clients and what is needed to facilitate the adoption of these cessation services within these settings. Removing barriers to tobacco-treatment provision for clients in substance-use treatment settings will include provider and organizational leader education to correct misperceptions, specialized training to equip providers with knowledge and skills for tobacco-use care, and resources to build organizational capacity. With the practical application of such things, clients in substance-use treatment settings should reap the benefits of improved tobacco intervention delivery.

## Figures and Tables

**Table 1:** Texas Healthcare Center Characteristics, Providers’ Perceptions on Concurrent Smoking/Tobacco Cessation and Non-Nicotine-Substance-Use Treatment, and Providers’ Provision of the 5A’s for Smoking Cessation.

Variables of Interest	All Centers
	% (n)
Healthcare Center Characteristics	
**Center type**	
Federally Qualified Health Center	10.84 (9)
Substance-use program within LMHA	6.02 (5)
LMHA overall	19.28 (16)
Stand-alone substance-use treatment center	63.86 (53)
**# of unique clients seen annually**	
50–200	32.10 (26)
201–1000	44.44 (36)
>1000	23.46 (19)
**# of full-time employees**	
1–50	61.45 (51)
>50	38.55 (32)
**Comprehensive tobacco-free workplace policy**	
Yes	48.19 (40)
No	51.81 (43)
Provider’s Perceptions on Concurrent Smoking/Tobacco Cessation and Non-Nicotine-Substance-Use Treatment *	
**In your opinion, what is the best point to encourage clients to stop smoking or using other tobacco products?**	
Never/It depends on the client/After 1 year of treatment	60.24 (50)
As soon as client begins treatment	39.76 (33)
**If a client has been in recovery from alcoholism for <6 months, quitting smoking would jeopardize their recovery. ⟡**	
Yes	45.78 (38)
No	54.22 (45)
Providers’ Provision of the 5A’s Smoking-cessation Intervention with Clients	
**Asks clients about cigarette smoking ◊**	
Yes	69.88 (58]
No	30.12 (25)
**Advises clients to quit smoking ◊**	
Yes	68.67 (57)
No	31.33 (26)
**Assesses client interest in quitting smoking ◊**	
Yes	62.65 (52)
No	37.35 (31)
**Assists a smoking quit attempt ◊**	
Yes	57.83 (48)
No	42.17 (35)
**Arranges a follow-up ◊**	
Yes	33.73 (28)
No	66.27 (55)

Note. N = 83 due to missing data on one or more of the variables of interest; # = number; LMHA = Local Mental Health Authority; ⟡: Yes = Neither agree nor disagree, agree, or strongly agree, No = Strongly disagree or Disagree; ◊: Yes = Always or Most of the time, No = Never, Sometimes, or About half the time; * = Complete distributions of the variables are available in Table S1 in the Supplemental files.

**Table 2:** Texas Substance-Use Treatment Providers’ Smoking Intervention Practices with Clients Relative to Their Perceptions of Concurrent Smoking/Tobacco Cessation and Non-Nicotine-Substance-Use Treatment.

		Adjusted Model			
		Estimate	SE	OR (95% CI)	*p*-Value
**Ask**	Intercept	2.234	0.654		0.001
	**Perceived Best Point to Encourage Clients to Stop Smoking or Using Other Tobacco Products (ref: As soon as client begins treatment)**	−1.634	0.620	0.195 (0.058, 0.657)	0.008
	Center Type FQHC (ref: SUTC)	0.983	0.894	2.672 (0.463, 15.409)	0.272
	Center Type LMHA (ref: SUTC)	1.565	1.752	4.781 (0.154, 148.271)	0.372
	Center Type Global LMHA (ref: SUTC)	−0.730	0.662	0.482 (0.132, 1.765)	0.270
	Comprehensive Tobacco-Free Workplace Policy (ref: No)	−0.766	0.558	0.465 (0.156, 1.389)	0.170
**Advise**	Intercept	2.607	0.701		<0.001
	**Perceived Best Point to Encourage Clients to Stop Smoking or Using Other Tobacco Products (ref: As soon as client begins treatment)**	−1.739	0.647	0.176 (0.049, 0.625)	0.007
	Center Type FQHC (ref: SUTC)	1.319	0.927	3.741 (0.608, 23.013)	0.155
	Center Type LMHA (ref: SUTC)	1.812	1.842	6.122 (0.166, 226.462)	0.325
	Center Type Global LMHA (ref: SUTC)	−0.847	0.671	0.429 (0.115, 1.599)	0.207
	Comprehensive Tobacco-Free Workplace Policy (ref: No)	−1.465	0.596	0.231 (0.072, 0.742)	0.014
**Assess**	Intercept	1.312	0.542		0.015
	**Perceived Best Point to Encourage Clients to Stop Smoking or Using Other Tobacco Products (ref: As soon as client begins treatment)**	−1.134	0.539	0.322 (0.112, 0.925)	0.035
	Center Type FQHC (ref: SUTC)	−0.489	0.783	0.614 (0.132, 2.846)	0.533
	Center Type LMHA (ref: SUTC)	1.676	1.665	5.346 (0.205, 139.606)	0.314
	Center Type Global LMHA (ref: SUTC)	−0.555	0.630	0.574 (0.167, 1.975)	0.379
	Comprehensive Tobacco-Free Workplace Policy (ref: No)	−0.017	0.514	0.983 (0.359, 2.691)	0.974
**Assist**	Intercept	0.725	0.488		0.137
	**Perceived Best Point to Encourage Clients to Stop Smoking or Using Other Tobacco Products (ref: As soon as client begins treatment)**	−0.848	0.497	0.428 (0.162, 1.134)	0.088
	Center Type FQHC (ref: SUTC)	0.670	0.799	1.955 (0.409, 9.350)	0.401
	Center Type LMHA (ref: SUTC)	0.755	1.088	2.129 (0.252, 17.961)	0.488
	Center Type Global LMHA (ref: SUTC)	−0.097	0.609	0.908 (0.275, 2.995)	0.874
	Comprehensive Tobacco-Free Workplace Policy (ref: No)	−0.022	0.488	0.979 (0.376, 2.545)	0.965
**Arrange**	Intercept	−0.667	0.490		0.174
	**Perceived Best Point to Encourage Clients to Stop Smoking or Using Other Tobacco Products (ref: As soon as client begins treatment)**	−0.696	0.505	0.498 (0.185, 1.342)	0.168
	Center Type FQHC (ref: SUTC)	0.136	0.818	1.145 (0.230, 5.695)	0.868
	Center Type LMHA (ref: SUTC)	1.028	0.979	2.795 (0.410, 19.043)	0.294
	Center Type Global LMHA (ref: SUTC)	0.016	0.632	1.016 (0.294, 3.507)	0.980
	Comprehensive Tobacco-Free Workplace Policy (ref: No)	0.661	0.511	1.936 (0.711, 5.274)	0.196
**Ask**	Intercept	1.435	0.466		0.002
	**Perceives that Quitting Smoking Jeopardizes Recovery from Alcoholism (ref: No)**	−0.975	0.518	0.377 (0.137, 1.040)	0.060
	Center Type FQHC (ref: SUTC)	0.825	0.899	2.281 (0.392, 13.287)	0.359
	Center Type LMHA (ref: SUTC)	1.874	1.666	6.512 (0.249, 170.382)	0.261
	Center Type Global LMHA (ref: SUTC)	−0.252	0.625	0.778 (0.228, 2.649)	0.688
	Comprehensive Tobacco-Free Workplace Policy (ref: No)	−0.571	0.531	0.565 (0.200, 1.599)	0.282
**Advise**	Intercept	1.555	0.480		0.001
	**Perceives that Quitting Smoking Jeopardizes Recovery from Alcoholism (ref: No)**	−0.703	0.524	0.495 (0.177, 1.381)	0.179
	Center Type FQHC (ref: SUTC)	0.966	0.905	2.628 (0.446, 15.476)	0.285
	Center Type LMHA (ref: SUTC)	1.868	1.686	6.475 (0.238, 176.478)	0.268
	Center Type Global LMHA (ref: SUTC)	−0.394	0.622	0.674 (0.199, 2.283)	0.526
	Comprehensive Tobacco-Free Workplace Policy (ref: No)	−1.115	0.538	0.328 (0.114, 0.942)	0.038
**Assess**	Intercept	0.723	0.405		0.074
	**Perceives that Quitting Smoking Jeopardizes Recovery from Alcoholism (ref: No)**	−0.536	0.479	0.585 (0.229, 1.496)	0.263
	Center Type FQHC (ref: SUTC)	−0.662	0.776	0.516 (0.113, 2.360)	0.394
	Center Type LMHA (ref: SUTC)	1.953	1.637	7.048 (0.285, 174.255)	0.233
	Center Type Global LMHA (ref: SUTC)	−0.269	0.613	0.764 (0.230, 2.543)	0.661
	Comprehensive Tobacco-Free Workplace Policy (ref: No)	0.123	0.498	1.131 (0.426, 3.000)	0.805
**Assist**	Intercept	0.525	0.396		0.185
	**Perceives that Quitting Smoking Jeopardizes Recovery from Alcoholism (ref: No)**	−0.895	0.479	0.409 (0.160, 1.044)	0.061
	Center Type FQHC (ref: SUTC)	0.708	0.814	2.031 (0.412, 10.011)	0.384
	Center Type LMHA (ref: SUTC)	1.151	1.096	3.163 (0.369, 27.107)	0.294
	Center Type Global LMHA (ref: SUTC)	0.222	0.617	1.248 (0.372, 4.186)	0.719
	Comprehensive Tobacco-Free Workplace Policy (ref: No)	0.004	0.486	1.004 (0.387, 2.604)	0.994
**Arrange**	Intercept	−0.866	0.417		0.038
	**Perceives that Quitting Smoking Jeopardizes Recovery from Alcoholism (ref: No)**	−0.635	0.506	0.53 (0.197, 1.430)	0.210
	Center Type FQHC (ref: SUTC)	0.123	0.814	1.131 (0.229, 5.578)	0.880
	Center Type LMHA (ref: SUTC)	1.326	0.995	3.768 (0.536, 26.465)	0.182
	Center Type Global LMHA (ref: SUTC)	0.246	0.640	1.279 (0.365, 4.487)	0.701
	Comprehensive Tobacco-Free Workplace Policy (ref: No)	0.689	0.508	1.992 (0.737, 5.388)	0.174

Note. N = 83 due to missing data on one or more of the variables of interest; FQHC = Federally Qualified Health Center; LMHA = Local Mental Health Authority; SUTC = substance-use treatment center; ref = reference group (coded at 0) for statistical comparison; SE = standard error; OR = Odds Ratio; CI = Confidence Interval; Ask, Advise, Assess, Assist, and Arrange are coded as: Yes (1) = Always or Most of the time, No (0) = Never, Sometimes, or About half the time.

**Table 3:** Qualitative Analysis: Summary of Themes and Findings.

Theme	Findings
Misconceptions about concurrent treatment of tobacco and substance use: smoking reduces stress and is a valuable coping mechanism.	Many participants espoused misconceptions that concurrent treatment of tobacco and substance use was detrimental to clients, in that it could jeopardize their substance-use recovery because: (1) Smoking reduces stress, and is a valuable coping mechanism. (2) Clients need to smoke to offset the added stress of trying to recover from non-nicotine substance use. These misconceptions were acknowledged by some participants as part of the culture of substance-use treatment. However, participants thought that providers could be trained to address tobacco dependence with their clients and provide them with alternatives to smoking.
“Blame the victim” mentality: clients are not interested in quitting smoking.	Most participants stated clients’ lack of interest in quitting smoking kept them from pursuing tobacco cessation with them. Only a few said that clients were genuinely interested in quitting smoking. However, some participants acknowledged that providers’ neglect in making time to address tobacco use and including it in clients’ treatment and recovery plan contributed to the failure to treat tobacco dependence at their center. Clients needed to initiate the receipt of tobacco-dependence treatment rather than providers, signaling the irrelevance of tobacco use to clients’ treatment and well-being
Attitudes towards smoking cessation: (a) devaluing of tobacco dependence as a serious addiction; (b) clients were overwhelmed by multiple social inequities and did not have the wherewithal to quit smoking.	The key attitudes participants expressed regarding smoking cessation included: (1) That smoking was not a serious addiction that required treatment; clients’ substance-use disorders took precedence over tobacco-cessation treatment. (2) Clients had contending and more important issues that needed addressing; once these issues were addressed, then smoking dependence could be tackled.
Limited resources for addressing tobacco dependence.	Participants have limited resources available to them to treat tobacco dependence, including a lack of: (1) Time: contending treatment priorities and high case loads; (2) Training: on how to treat tobacco dependence; (3) Staff: to dedicate to treat tobacco dependence; (4) Funding: that could be devoted to smoking cessation. Participants noted the need for specialized training to treat tobacco dependence as essential to increase organizational capacity in order to address client tobacco and substance use concurrently.

## Data Availability

The data presented in this study are available upon request from the corresponding author. The data are not publicly available due to funder restrictions and because outcome papers are still being reported from the dataset.

## Supplementary Materials

The following supporting information can be downloaded at: https://www.mdpi.com/article/10.3390/ijerph20075260/s1, Table S1: Distribution of Providers’ Perceptions about Concurrent Smoking/Tobacco Cessation and Non-nicotine Substance Use Treatment; Table S2: Providers’ Perceptions on Concurrent Smoking/Tobacco Cessation and Non-nicotine Substance Use Treatment, and Providers’ Provision of the 5A’s for Smoking Cessation, by Center Type; Table S3: Providers’ Perceptions on Concurrent Smoking/Tobacco Cessation and Non-nicotine Substance Use Treatment, and Providers’ Provision of the 5A’s for Smoking Cessation, by Numbers of Unique Clients Seen Annually; Table S4: Providers’ Perceptions on Concurrent Smoking/Tobacco Cessation and Non-nicotine Substance Use Treatment, and Providers’ Provision of the 5A’s for Smoking Cessation, by Number of Full-time Employees; Table S5: Providers’ Perceptions on Concurrent Smoking/Tobacco Cessation and Non-nicotine Substance Use Treatment, and Providers’ Provision of the 5A’s for Smoking Cessation, by Comprehensive Tobacco Free Workplace Policy Implementation; Table S6: Texas Substance Use Treatment Providers’ Smoking Intervention Practices with Clients Relative to Their Perceptions of Concurrent Smoking/Tobacco Cessation and Non-Nicotine Substance Use Treatment (Unadjusted Models).
