# Supplementary material for: Influence of Provider and Leader Perspectives about Concurrent Tobacco-Use Care during Substance-Use Treatment on Their Tobacco Intervention Provision with Clients: A Mixed-Methods Study
Source: Int J Environ Res Public Health. 2023 Mar 24;20(7):5260. doi: 10.3390/ijerph20075260 (PMC10094458; doi:10.3390/ijerph20075260)
Supplement: Supplementary file 1 [file ijerph-20-05260-s001.zip › ijerph-2149907-supplementary.pdf]

Table S1. Distribution of Providers' Perceptions about Concurrent Smoking/Tobacco Cessation and Non-nicotine Substance Use Treatment.

Provider's Perceptions on Concurrent Smoking/Tobacco Cessation and Non-nicotine Substance Use Treatment	All Centers % (n)
**In your opinion, what is the best point to encourage clients to stop smoking or using other tobacco products?**	
As soon as client begins treatment	39.76 (33)
After 1 year of treatment	1.20 (1)
It depends on the client	57.83 (48)
Never	1.20 (1)
**If a client has been in recovery from alcoholism for <6 months, quitting smoking would jeopardize their recovery.**	
Strongly disagree	21.69 (18)
Disagree	32.53 (27)
Neither agree nor disagree	30.12 (25)
Agree	9.64 (8)
Strongly agree	6.02 (5)

Note. N=83 due to missing data on one or more variables of interest.

Table S2. Providers' Perceptions on Concurrent Smoking/Tobacco Cessation and Non-nicotine Substance Use Treatment, and Providers' Provision of the 5A's for Smoking Cessation, by Center Type.

Variables of Interest	All Centers	Center Type				p-value§
		FQHC	SUP within LMHA	LMHA overall	Stand-alone SUTC	
	% (n)					
Provider's Perceptions on Concurrent Smoking/Tobacco Cessation and Non-nicotine Substance Use Treatment						
**In your opinion, what is the best point to encourage clients to stop smoking or using other tobacco products?**						0.1128
Never/It depends on the client/After 1 year of treatment	60.24 (50)	88.89 (1)	40.00 (2)	43.75 (7)	62.26 (33)	
As soon as client begins treatment	39.76 (33)	11.11 (1)	60.00 (3)	56.25 (9)	37.74 (20)	
**If a client has been in recovery from alcoholism for <6 months, quitting smoking would jeopardize their recovery. ✧**						0.2526
Yes	45.78 (38)	66.67 (6)	60.00 (3)	56.25 (9)	37.74 (20)	
No	54.22 (45)	33.33 (3)	40.00 (2)	43.75 (7)	62.26 (33)	
Providers' Provision of the 5A's Smoking Cessation Intervention with Clients						
**Asks clients about cigarette smoking◊**						0.3448
Yes	69.88 (58)	77.78 (7)	100.00 (5)	56.25 (9)	69.81 (37)	
No	30.12 (25)	22.22 (2)	0.00 (0)	43.75 (7)	30.19 (16)	
**Advises clients to quit smoking◊**						0.1861
Yes	68.67 (57)	77.78 (7)	100.00 (5)	50.00 (8)	69.81 (37)	
No	31.33 (26)	22.22 (2)	0.00 (0)	50.00 (8)	30.19 (16)	
**Assesses client interest in quitting smoking◊**						0.2074
Yes	62.65 (52)	44.44 (4)	100.00 (5)	56.25 (9)	64.15 (34)	
No	37.35 (31)	55.56 (5)	0.00 (0)	43.75 (7)	35.85 (19)	
**Assists a smoking quit attempt◊**						0.7466
Yes	57.83 (48)	66.67 (6)	80.00 (4)	56.25 (9)	54.72 (29)	

No	42.17 (35)	33.33 (3)	20.00 (1)	43.75 (7)	45.28 (24)	
**Arranges a follow-up◊**						0.6064
Yes	33.73 (28)	33.33 (3)	60.00 (3)	37.50 (6)	30.19 (16)	
No	66.27 (55)	66.67 (6)	40.00 (2)	62.50 (10)	69.81 (37)	

Note. N=83 due to missing data on one or more of the variables of interest; LMHA=Local Mental Health Authority; ✧: Yes=Neither agree nor disagree, agree, or strongly agree, No=Strongly disagree or Disagree; ◊: Yes=Always or Most of the time, No=Never, Sometimes, or About half the time; §: p-value of Fisher's exact test.

Table S3. Providers' Perceptions on Concurrent Smoking/Tobacco Cessation and Non-nicotine Substance Use Treatment, and Providers' Provision of the 5A's for Smoking Cessation, by Numbers of Unique Clients Seen Annually.

Variables of Interest	All Centers	# of Unique Clients Seen Annually			p-value§
		50-200	201-1,000	>1,000	
	% (n)				
Provider's Perceptions on Concurrent Smoking/Tobacco Cessation and Non-nicotine Substance Use Treatment					
**In your opinion, what is the best point to encourage clients to stop smoking or using other tobacco products?**					0.0621
Never/It depends on the client/After 1 year of treatment	59.26 (48)	61.54 (16)	69.44 (25)	36.84 (7)	
As soon as client begins treatment	40.74 (33)	38.46 (10)	30.56 (11)	63.16 (12)	
**If a client has been in recovery from alcoholism for <6 months, quitting smoking would jeopardize their recovery. ✧**					0.7386
Yes	45.68 (37)	46.15 (12)	41.67 (15)	52.63 (10)	
No	54.32 (45)	53.85 (14)	58.33 (21)	47.37 (9)	
Providers' Provision of the 5A's Smoking Cessation Intervention with Clients					
**Asks clients about cigarette smoking◊**					0.9366
Yes	70.37 (57)	69.23 (18)	69.44 (25)	73.68 (14)	
No	29.63 (25)	30.77 (8)	30.56 (11)	26.32 (5)	
**Advises clients to quit smoking◊**					0.8450
Yes	69.14 (56)	65.38 (17)	72.22 (26)	68.42 (13)	
No	30.86 (25)	34.62 (9)	27.78 (10)	31.58 (6)	
**Assesses client interest in quitting smoking◊**					0.3585
Yes	64.20 (52)	53.85 (14)	66.67 (24)	73.68 (14)	
No	35.80 (29)	46.15 (12)	33.33 (12)	26.32 (5)	
**Assists a smoking quit attempt◊**					0.1602
Yes	56.79 (46)	42.31 (11)	66.67 (24)	57.89 (11)	

No	43.21 (35)	57.69 (15)	33.33 (12)	42.11 (8)	
**Arranges a follow-up◊**					0.4885
Yes	32.10 (26)	23.08 (6)	36.11 (13)	36.84 (7)	
No	67.90 (55)	76.92 (20)	63.89 (23)	63.16 (12)	

Note. N=83 due to missing data on one or more of the variables of interest; LMHA=Local Mental Health Authority; ✧: Yes=Neither agree nor disagree, agree, or strongly agree, No=Strongly disagree or Disagree; ◊: Yes=Always or Most of the time, No=Never, Sometimes, or About half the time; §: p-value of Chi-square test.

Table S4. Providers' Perceptions on Concurrent Smoking/Tobacco Cessation and Non-nicotine Substance Use Treatment, and Providers' Provision of the 5A's for Smoking Cessation, by Number of Full-time Employees.

Variables of Interest	All Centers	Number of Full-time Employees		p-value§
		1-50	>50	
	% (n)			
Provider's Perceptions on Concurrent Smoking/Tobacco Cessation and Non-nicotine Substance Use Treatment				
**In your opinion, what is the best point to encourage clients to stop smoking or using other tobacco products?**				0.8984
Never/It depends on the client/After 1 year of treatment	60.24 (50)	60.78 (31)	59.38 (19)	
As soon as client begins treatment	39.76 (33)	39.22 (20)	40.63 (13)	
**If a client has been in recovery from alcoholism for <6 months, quitting smoking would jeopardize their recovery. ✧**				0.5413
Yes	45.78 (38)	43.14 (22)	50.00 (16)	
No	54.22 (45)	56.86 (29)	50.00 (16)	
Providers' Provision of the 5A's Smoking Cessation Intervention with Clients				
**Asks clients about cigarette smoking◊**				0.2457
Yes	69.88 (58)	74.51 (38)	62.50 (20)	
No	30.12 (25)	25.49 (13)	37.50 (12)	
**Advises clients to quit smoking◊**				0.0532
Yes	68.67 (57)	76.47 (39)	56.25 (18)	
No	31.33 (26)	23.53 (12)	43.75 (14)	
**Assesses client interest in quitting smoking◊**				0.9821
Yes	62.65 (52)	62.75 (32)	62.50 (20)	
No	37.35 (31)	37.25 (19)	37.50 (12)	
**Assists a smoking quit attempt◊**				0.8215
Yes	57.83 (48)	56.86 (29)	59.38 (19)	
No	42.17 (35)	43.14 (22)	40.63 (13)	

**Arranges a follow-up◊**				0.9222
Yes	33.73 (28)	33.33 (17)	34.38 (11)	
No	66.27 (55)	66.67 (34)	65.63 (21)	

Note. N=83 due to missing data on one or more of the variables of interest; LMHA=Local Mental Health Authority; ✧: Yes=Neither agree nor disagree, agree, or strongly agree, No=Strongly disagree or Disagree; ◊: Yes=Always or Most of the time, No=Never, Sometimes, or About half the time; §: p-value of Chi-square test.

Table S5. Providers' Perceptions on Concurrent Smoking/Tobacco Cessation and Non-nicotine Substance Use Treatment, and Providers' Provision of the 5A's for Smoking Cessation, by Comprehensive Tobacco Free Workplace Policy Implementation.

Variables of Interest	All Centers	Comprehensive Tobacco Free Workplace Policy		p-value§
		Yes	No	
	% (n)			
Provider's Perceptions on Concurrent Smoking/Tobacco Cessation and Non-nicotine Substance Use Treatment				
**In your opinion, what is the best point to encourage clients to stop smoking or using other tobacco products?**				0.1646
Never/It depends on the client/After 1 year of treatment	60.24 (50)	52.50 (21)	67.44 (29)	
As soon as client begins treatment	39.76 (33)	47.50 (19)	32.56 (14)	
**If a client has been in recovery from alcoholism for <6 months, quitting smoking would jeopardize their recovery. ✧**				0.5626
Yes	45.78 (38)	42.50 (17)	48.84 (21)	
No	54.22 (45)	57.50 (23)	51.16 (22)	
Providers' Provision of the 5A's Smoking Cessation Intervention with Clients				
**Asks clients about cigarette smoking◊**				0.3500
Yes	69.88 (58)	65.00 (26)	74.42 (32)	
No	30.12 (25)	35.00 (14)	25.58 (11)	
**Advises clients to quit smoking◊**				0.0343
Yes	68.67 (57)	57.50 (23)	79.07 (34)	
No	31.33 (26)	42.50 (17)	20.93 (9)	
**Assesses client interest in quitting smoking◊**				0.9782
Yes	62.65 (52)	62.50 (25)	62.79 (27)	
No	37.35 (31)	37.50 (15)	37.21 (16)	
**Assists a smoking quit attempt◊**				
Yes	57.83 (48)	60.00 (24)	55.81 (24)	0.6996

No	42.17 (35)	40.00 (16)	44.19 (19)	
**Arranges a follow-up◊**				0.1033
Yes	33.73 (28)	42.50 (17)	25.58 (11)	
No	66.27 (55)	57.50 (23)	74.42 (32)	

Note. N=83 due to missing data on one or more of the variables of interest; LMHA=Local Mental Health Authority; ✧: Yes=Neither agree nor disagree, agree, or strongly agree, No=Strongly disagree or Disagree; ◊: Yes=Always or Most of the time, No=Never, Sometimes, or About half the time; §: p-value of Chi-square test.

Table S6. Texas Substance Use Treatment Providers' Smoking Intervention Practices with Clients Relative to Their Perceptions of Concurrent Smoking/Tobacco Cessation and Non-Nicotine Substance Use Treatment (Unadjusted Models).

		Unadjusted Model			
		Estimate	SE	OR (95% CI)	p-value
**Ask**	Intercept	1.723	0.485		<0.001
	**Perceived Best Point to Encourage Clients to Stop Smoking or Using Other Tobacco Products (ref: As soon as client begins treatment)**	-1.317	0.565	0.268 (0.089, 0.810)	0.020
**Advise**	Intercept	1.504	0.451		0.001
	**Perceived Best Point to Encourage Clients to Stop Smoking or Using Other Tobacco Products (ref: As soon as client begins treatment)**	-1.099	0.536	0.333 (0.117, 0.953)	0.040
**Assess**	Intercept	1.312	0.426		0.002
	**Perceived Best Point to Encourage Clients to Stop Smoking or Using Other Tobacco Products (ref: As soon as client begins treatment)**	-1.232	0.511	0.292 (0.107, 0.795)	0.016
**Assist**	Intercept	0.833	0.379		0.028
	**Perceived Best Point to Encourage Clients to Stop Smoking or Using Other Tobacco Products (ref: As soon as client begins treatment)**	-0.833	0.473	0.435 (0.172, 1.098)	0.078
**Arrange**	Intercept	-0.182	0.350		0.602
	**Perceived Best Point to Encourage Clients to Stop Smoking or Using Other Tobacco Products (ref: As soon as client begins treatment)**	-0.864	0.476	0.422 (0.166, 1.071)	0.069
**Ask**	Intercept	1.253	0.359		<0.001
	**Perceives that Quitting Smoking Jeopardizes Recovery from Alcoholism (ref: No)**	-0.825	0.489	0.438 (0.168, 1.141)	0.091
**Advise**	Intercept	1.012	0.337		0.003
	**Perceives that Quitting Smoking Jeopardizes Recovery from Alcoholism (ref: No)**	-0.473	0.476	0.623 (0.245, 1.585)	0.321

**Assess**	Intercept	0.795	0.322		0.014
	**Perceives that Quitting Smoking Jeopardizes Recovery from Alcoholism (ref: No)**	-0.584	0.458	0.558 (0.227, 1.370)	0.203
**Assist**	Intercept	0.693	0.316		0.028
	**Perceives that Quitting Smoking Jeopardizes Recovery from Alcoholism (ref: No)**	-0.798	0.453	0.450 (0.185, 1.094)	0.078
**Arrange**	Intercept	-0.405	0.304		0.183
	**Perceives that Quitting Smoking Jeopardizes Recovery from Alcoholism (ref: No)**	-0.624	0.478	0.536 (0.210, 1.367)	0.192

Notes. N=83 due to missing data on one or more of the variables of interest; FQHC = Federally Qualified Health Center; LMHA = Local Mental Health Authority; SUTC = substance use treatment center; ref = reference group (coded at 0) for statistical comparison; SE = standard error; OR = Odds Ratio; CI = Confidence Interval; Ask, Advise, Assess, Assist, and Arrange are coded as: Yes (1)=Always or Most of the time, No (0)=Never, Sometimes, or About half the time.
